# Fine-Tuned Large Language Models for High-Accuracy Prediction of Band Gap and Stability in Transition Metal Sulfides

**DOI:** 10.3390/ma18163793

**Published:** 2025-08-13

**Authors:** Zimo Zhao, Lin Hu, Honghui Wang

**Affiliations:** 1 Demonstrative Software School, College of Computer Science and Cyber Security, Chengdu University of Technology, Chengdu 610059, China; zhao.zimo@student.zy.cdut.edu.cn; 2 College of Physics and Electronics Engineering, Sichuan Normal University, Chengdu 610101, China; hl22662023@163.com

**Keywords:** catalytic activity, large language models, material property prediction, fine-tuning, traditional models, transition metal sulfides

## Abstract

This study presents a fine-tuned Large Language Model approach for predicting band gap and stability of transition metal sulfides. Our method processes textual descriptions of crystal structures directly, eliminating the need for complex feature engineering required by traditional ML and GNN approaches. Using a strategically selected dataset of 554 compounds from the Materials Project database, we fine-tuned GPT-3.5-turbo through nine consecutive iterations. Performance metrics improved significantly, with band gap prediction R^2^ values increasing from 0.7564 to 0.9989, while stability classification achieved F1 > 0.7751. The fine-tuned model demonstrated superior generalization ability compared to both GPT-3.5 and GPT-4.0 models, maintaining high accuracy across diverse material structures. This approach is particularly valuable for new material systems with limited experimental data, as it can extract meaningful features directly from text descriptions and transfer knowledge from pre-training to domain-specific tasks without relying on extensive numerical datasets.

## 1. Introduction

Owing to the electronic character of transition metal sulfides, these substances hold a central position in catalysis, energy storage, and even photovoltaic technology [1,2]. Their catalytic activity determination, nevertheless, is still plenty of hard work, requiring manual and meticulous handling. Among them, band gap and stability are the most important properties that control the catalytic effect. These two factors control important structural characteristics, such as the development of active centers and the effectiveness of controlling the efficient electron transfer catalyzed by transition metal sulfides [3]. Researchers have recently published developments in the use of machine learning (ML) techniques [4] to predict material properties like band gap [5,6], which can greatly simplify the search for specific materials.

Traditional approaches for investigating transition metal sulfide properties are computational and experimental. Experimentally, the band gap calculations typically include techniques like photoluminescence spectroscopy and UV-visible absorption spectroscopy [7]. However, these experimental techniques are limited to materials that have been synthesized exclusively and not to theoretically conceptualized compounds whose stability could only be predicted theoretically. For computational purposes, Density Functional Theory (DFT) has become the foundation for investigating the electronic structure of materials made up of known as well as theoretically stable compounds. DFT performs well in simulating electronic properties, including band structures and band gaps [8,9]. Its usage in experimental high-throughput material screening is, however, hampered by multiple computational requirements and resource-intensive processes, particularly with complex systems [10].

To solve the computational limitations of DFT, machine learning methods have become a powerful alternative tool, which is capable of accelerating materials property prediction while maintaining reasonable accuracy. Traditional machine learning models such as Random Forest (RF) [11] and Support Vector Machines (SVM) [12,13,14] are usually used to predict band gap and structural stability. Unlike complex physical models, these approaches offer better adaptability and significantly enhance the efficiency of material screening [15,16,17]. However, when dealing with large-scale and high-dimensional data, they face significant challenges and are often difficult to train and interpret. They may be restricted by model and algorithmic architectures for complex tasks, which render generalization capacity weak, require more computational resources, and exhibit unstable performance on unseen data.

The latest advancements in deep learning, especially Graph Neural Networks (GNNs) [18,19,20,21,22], have enhanced feature extraction ability by seamlessly learning representations from the graph structure of materials. This method reduces the reliance on elaborately designed features and is more preferable for heterogeneous material data of different sizes and complexities. In terms of material property prediction, GNN can handle atomic structure diagrams and extract complex relationships between atoms or parts. On the other hand, the construction of GNNs requires a large amount of labeled data, generates high costs related to computing, and often lacks clear and meaningful features, which poses a serious limitation to their wide application in the field of materials science.

Despite these advances, three technical gaps remain unresolved. (i) High-fidelity DFT still dominates the creation of labelled datasets, which limits the chemical diversity that can be covered in a reasonable wall-clock time. (ii) Descriptor-driven ML models—while fast—depend on handcrafted features whose transferability from one crystal family to another is difficult to guarantee. (iii) State-of-the-art GNNs alleviate manual featurization but rely on tens of thousands of labeled structures to avoid over-smoothing and over-squashing, a requirement rarely met in niche catalyst sub-domains such as transition-metal sulfides. These bottlenecks motivate a paradigm that can learn from existing unstructured text materials and operate in a data-efficient regime.

Recent breakthroughs in natural-language processing (NLP) [23] have produced large Language models (LLMs) [24,25] with billions of parameters that can be instruction-tuned to specialized domains. Unlike graph-based models that take atomic coordinates as input, an LLM ingests plain text. It, therefore, becomes possible to exploit the terabytes of crystallographic prose already produced by ICSD entries, Materials Project “structure descriptions”, OQMD [26] notebook annotations, and literature corpora mined by tools such as ChemDataExtractor [27]. By casting each material as a short narrative—automatically generated with robocrystallographer [28] or scraped from papers—LLMs can learn latent structure–property relationships implicit in human language. This text-native formulation removes the need for elaborate descriptor engineering, retains chemical interpretability through attention weights, and can be fine-tuned with only a few hundred labeled samples, as demonstrated in this work.

To our knowledge, prior applications of LLMs in materials science have focused on retrieval or chat-style assistance rather than quantitative property prediction. Here, we take a different route: GPT-3.5-turbo is fine-tuned end-to-end on 554 transition-metal-sulfide entries, each paired with numerically accurate band-gap and thermodynamic-stability labels computed in the Materials Project. This setting allows a controlled comparison against: (i) descriptor-based ML (Random Forest, SVM, XGBoost, LightGBM) and (ii) coordinate-based deep GNNs reported in the literature. Our results show that a text-only LLM, when carefully fine-tuned, can match or exceed the predictive accuracy of these baselines while using <1000 labeled points—two orders of magnitude fewer than typical GNN benchmarks such as MatBench [29].

Integrating cutting-edge NLP techniques with materials science is a notable advancement in the capabilities for predicting materials. Recent developments have enhanced the prediction of structural properties for transformer-based models like AlloyBERT [30] and AMGPT [31]. Successful applications of the newer approaches include band gap prediction and assessing the stability of transition metal sulfides [32,33], which present novel solutions to traditional methods of feature engineering. Attention mechanisms inherent to the implemented transformer architectures [34] assist in interpretability as they enrich descriptions of critical materials and offer deeper understanding regarding the prediction.

Unlike previous studies that relied heavily on numerical feature engineering, this work pioneers the application of fine-tuned LLMs to predict catalytic properties directly from textual material descriptions. Our research goal is to develop an automated workflow to predict target characteristics related to the catalytic activity of transition metal sulfides so that accelerating the discovery of target materials. This study proposes an innovative approach that integrates the Materials Project database [35] API for data collection with the robocrystallographer [28] for feature extraction (an automated tool for generating text descriptions of crystal structures). By using fine-tuned language models, our approach processes text-based representations of materials directly while eliminating the need for extensive preprocessing of both numerical and non-numerical features. The framework accurately predicts the band gap and stability of transition metal sulfides. This dual predictive ability not only deepens the fundamental understanding of these materials but also accelerates the discovery of new stable compounds with ideal electronic properties.

## 2. Materials and Methods

**Figure 1:**
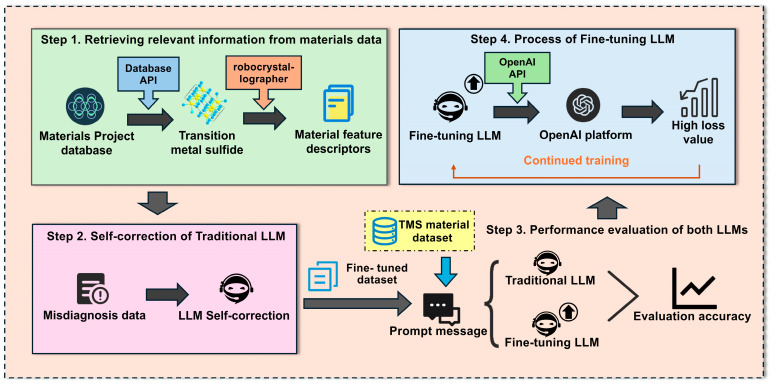
Flowchart for developing a domain-specific LLM tailored to predict band gap and stability of transition metal sulfides.

Our research methodology follows a structured workflow for developing and evaluating a domain-specific Large Language Model (LLM) optimized for transition metal sulfide analysis, as illustrated in Figure 1. This comprehensive framework consists of four interconnected steps: In Step 1, data acquisition begins with extracting transition metal sulfide data from the Materials Project database API using specific chemical criteria. The robocrystallographer [28] then converts crystallographic structures into standardized textual descriptions, generating material feature descriptors that capture atomic arrangements, bond properties, and electronic characteristics in natural language format. Step 2 implements a self-correction process for traditional LLMs by identifying and addressing misdiagnosis data through verification protocols that cross-validate property predictions against established computational principles. This ensures training data reliability, which is crucial for specialized scientific domains. Step 3 conducts performance evaluation of both traditional and fine-tuned LLM variants using standardized prompt templates and metrics ($R^{2}$, RMSE, F1 score) to quantify differences between general-purpose and domain-specialized models. Step 4 employs iterative fine-tuning on the OpenAl with API version dated 30 May 2024 using supervised learning with structured JSONL format training examples. The process includes progressive multi-iteration training through loss tracking and targeted improvement of high-loss data points. Each iteration aims to minimize loss values while preserving generalization capabilities. This systematic approach enabled the development of a specialized model for predicting catalytic properties of transition metal sulfides with high accuracy, overcoming the limitations of traditional methods in this complex materials domain.

### 2.1. High-Quality Dataset Construction

**Figure 2:**
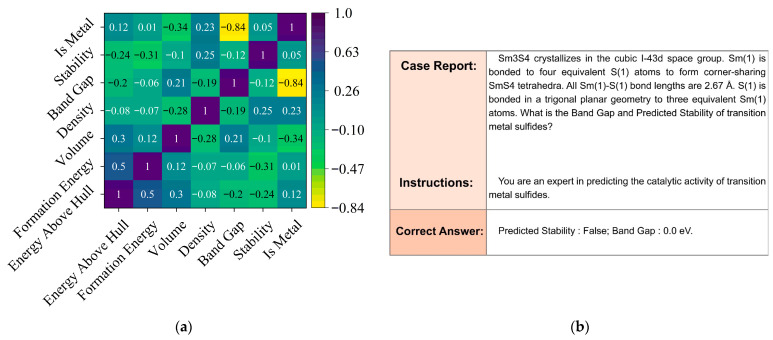
(**a**) Statistical Evaluation of Feature Correlations in Transition Metal Sulfide Datasets for traditional ML; (**b**) Case Analysis of Transition Metal Sulfide Property Prediction Using Fine-tuned Models.

As shown in the first step of Figure 1, we extracted relevant material data from the Materials Project database through its API, while using the robocrystallographer [28] to construct transition metal sulfide structures and generate associated material feature descriptors. The dataset was extracted using specific API parameters: transition metals (Sc-Zn, Y-Cd, La-Hg) combined with sulfur, formation energy below 500 eV/atom, and energy above hull < 150 eV/atom for thermodynamic stability. From 729 initial compounds, we eliminated 175 samples based on rigorous criteria: incomplete electronic structure data (61), unconverged relaxations with forces exceeding 0.05 eV/Å (43), disordered structures with partial occupancies (37), inconsistent band gap calculations between different computational methods (24), and unphysical bond configurations (10). The resulting 554 compounds formed our final dataset for model training and evaluation. In our research, we extend data-efficient approaches into the domain of transition metal sulfide catalysts, building upon established precedents in materials informatics. We adopted Wu et al.’s [36] transfer learning techniques that achieved 40% MAE reduction (from 0.0327 to 0.0204 W/(m·K)) with only 28 homopolymer samples, and implemented Wang et al.’s [37] insight that carefully selected high-quality training data can outperform larger noisy datasets. As illustrated in Figure 2a, our statistical evaluation reveals important feature correlations in transition metal sulfide datasets, where properties like energy above hull, formation energy, and band gap exhibit complex interdependencies that traditional machine learning approaches must navigate. In particular, the Pearson map reveals three illustrative patterns that underscore the complexity of the descriptor space. First, Energy Above Hull is moderately correlated with Formation Energy (r ≈ 0.50) yet anti-correlated with the binary Stability label (r ≈ −0.31); meanwhile, Formation Energy itself shows only a weak link to Stability (r ≈ 0.12). This triangular relationship implies that metastability cannot be inferred from any single energetic metric alone. Second, Band Gap displays a strong negative correlation with the categorical variable Is Metal (r ≈ −0.84), but its correlation with the continuous Density is weak (r ≈ −0.19), indicating that electronic character decouples from simple packing effects. Third, Volume correlates positively with Energy Above Hull (r ≈ 0.30) yet negatively with Band Gap (r ≈ −0.28), suggesting competing steric and electronic influences on lattice expansion. Such cross-dependencies mean that descriptors affecting the same target can interact in opposite ways, creating highly non-linear decision boundaries that motivate our shift from hand-crafted features to representation-learning via large language models. By curating 554 entries and implementing optimized techniques for catalytic materials, we achieved substantial improvements in predictive accuracy (R^2^ increasing from 0.7564 to 0.9989). Figure 2b demonstrates our fine-tuned model’s capability through a case analysis of SmS4, accurately predicting its band gap (0.0 eV) and predicted stability (False) based on its crystalline structure and bonding characteristics. The research indicates that in catalytic materials science, experimental validation is resource-intensive, property relationships require complex modeling, and strategic data selection can yield better results rather than simply expanding the dataset size, establishing a materials informatics paradigm, and maximizing value from limited but high-quality experimental data.

### 2.2. Traditional Language Models

Following the second step in Figure 1, we implemented a self-correction process for traditional LLMs to deal with misdiagnosis data, thus enhancing the robustness of the model. For benchmarking, we selected four traditional machine learning algorithms based on their complementary strengths in materials property prediction: RF for handling mixed feature types and robust performance with limited data, SVM for effectiveness with non-linear relationships and class imbalance, Extreme Gradient Boosting (XGBoost) for gradient-boosted performance with regularization capabilities and Light Gradient Boosting (LightGBM) for computational efficiency with comparable accuracy. This comprehensive evaluation addresses a critical challenge in materials science with significant implications for various industrial applications, focusing on band gap and predicted stability as crucial indicators of catalytic potential. To demonstrate that these four methods indeed provide the best accuracy–efficiency trade-off for our dataset, we additionally benchmarked four other popular algorithms (MLP-Regressor, GradientBoosting, k-NN, and Gaussian Process); the complete results are presented in Section S3 of the Supporting Information.

Our data preprocessing phase involved several crucial steps to ensure quality and compatibility, including eliminating irrelevant columns such as ‘Space Group Symbol’ and ‘Crystal System’, applying one-hot encoding to the ‘Formula’ column, and label encoding to the ‘Magnetic Ordering’ column. The ‘Total Magnetization’ column was converted to numeric values with missing entries filled with zeros, while potential infinity values and NaN entries were addressed to maintain data integrity.

Each model was configured with optimized parameters determined through grid search: RF (n_estimators = [100, 200, 300], max_depth = [10, 20, 30], min_samples_split = [2, 5, 10]), SVM (kernel = ‘rbf’, c = 1.0, epsilon = 0.1), XGBoost (learning_rate = 0.1, max_depth = 5, n_estimators = 100) and LightGBM (num_leaves = [20–100], feature_fraction = [0.5–1.0], bagging_fraction = [0.5–1.0], max_depth = [5–20]). All models underwent identical preprocessing with StandardScaler normalization and feature selection through RFE. We benchmarked the number of retained descriptors from 5 to 50 (step = 5) and found that 20 descriptors maximized the cross-validated R^2^ across all four algorithms while maintaining the simplicity of the model (see Figure S1 and Table S1 in the Supporting Information). The dataset was subsequently divided into training (80%) and testing (20%) sets, with each model undergoing hyperparameter tuning using GridSearchCV with 3-fold cross-validation.

In the evaluation stage, two target variables were treated with different metrics: R^2^, RMSE, and MSE for band gap prediction (considered a regression task), while accuracy, F1 score, and recall were used for predicted stability classification to evaluate performance more completely. The processes of data preprocessing, feature extraction, and system model optimization constitute an advanced framework for benchmarking classical machine learning algorithms concerning estimating the performance of materials, which sets the standard for future work in materials informatics and catalysis.

### 2.3. Prompt-Only ChatGPT Approach

We explored prompt-only interaction with large language models as a baseline comparison approach. This technique allows users to leverage simple prompts to effectively interact with LLMs without additional training. In zero-shot scenarios [38], the model generates predictions using only descriptive prompts and pre-existing knowledge. For enhancing performance, we implemented few-shot learning approaches, where the model uses selected examples to improve understanding and response accuracy. We carefully curated diverse sample prompts to maximize model performance across different material types and structural complexities. Our experiments interacted with ChatGPT using OpenAI API keys, employing both GPT-3.5 and GPT-4.0 models for comparative evaluation against our fine-tuned variants.

### 2.4. Fine-Tuning of Large Language Models

As illustrated in steps 3 and 4 of Figure 1, we conducted a performance evaluation of both traditional and fine-tuned LLM variants, leveraging the transition metal sulfides material dataset to formulate prompt messages and assess evaluation accuracy, followed by an iterative fine-tuning process employing the OpenAI for continued training with the objective of minimizing the loss value. The GPT-3.5-turbo fine-tuning employed a progressive nine-iteration approach (1st-FT through 9th-FT), each using supervised learning with three epochs per iteration. We implemented early stopping with a maximum wait time of 20000 steps to balance convergence and training efficiency. Throughout the process, we continuously monitored performance through comprehensive metrics including training loss, validation loss, and full validation loss, with periodic evaluation on a held-out test set. During both the classical-model benchmarks and the GPT-3.5-turbo fine-tuning, we explicitly minimized task-specific loss functions to guide parameter updates. For the continuous band-gap target the objective was the mean-squared error (MSE), defined as $L_{MSE}=\left(\frac{1}{N}\right)\sum_{i=1}^{N}{\left({\hat{g}}_{i}-g_{i}\right)}^{2}$, while for the binary stability label, we minimized the binary cross-entropy (BCE), $L_{BCE}=-(\frac{1}{N})\sum_{i=1}^{N}\left[y_{i}ln{\hat{p}}_{i}+\left(1-y_{i}\right)\ln\left(1-{\hat{p}}_{i}\right)\right]$. Inside the OpenAI supervised-fine-tuning loop, the network weights were updated with the standard token-level negative log-likelihood (NLL), $L_{NLL}=-\sum_{t}lnP_{\theta }(w_{t}|w_{<t})$; after each epoch the generated answers were parsed and their numerical components were evaluated with the same MSE or BCE on the validation set. These objectives were also used as scoring metrics for the GridSearchCV hyper-parameter optimization of the four classical algorithms (RF, SVM, XGBoost, and LightGBM), ensuring a consistent optimization criterion across all models discussed in this work. The training protocol utilized the OpenAI API with custom configuration to ensure optimal model performance. For robust performance tracking, we developed a pattern recognition system that extracted and visualized loss metrics from event logs, enabling precise identification of training trends. For convenience, we later refer to runs 1–3, 4–6, and 7–9 as early, mid, and late blocks because the loss curves display three clearly separated slopes (Section S1). These blocks emerged a posteriori and were not enforced through extra labels or tasks. Our framework also identified high-loss data points (those exceeding a threshold of 0.5 for training loss and 1.0 for validation loss) for targeted improvement in subsequent iterations, because these cut-offs correspond to the 95th percentile of the loss distributions across all nine fine-tuning iterations (see Section S5 in supporting information). As shown in Section S1, loss values decreased substantially across iterations, with the most significant improvements occurring in iterations 4–6 (post hoc observation, no extra objectives were applied in these iterations).

This fine-tuned dataset uses JSONL format, where each entry contains three elements: system, user, and assistant. The user role always contains the following prompt: “What is the band gap and predicted stability of transition metal sulfides?” The assistant role provides the corresponding values for each compound in the following format: {“Band gap: XXX eV; Predicted stability: XXX”}. This standardized structure efficiently trains language models [39] by maintaining consistent question-answer patterns about material properties, enabling clear mapping between queries and catalytic characteristics for each compound.

## 3. Results and Discussion

### 3.1. Description of Fine-Tuned Datasets for Transition Metal Sulfides

The study utilized transition metal sulfide data from the Materials Project database [35], covering 49 elements in groups III–XII (including lanthanides and actinides). The initial dataset of 729 entries was refined to 554 through the removal of 175 unstable compounds. The JSONL-formatted dataset contains two key features: band gap (0–3.03 eV) and predicted stability (binary: 0/1). Band gap determines electronic conductivity and charge transfer, while stability reflects operational durability of catalytic activity. The 8:2 training-test split ensures optimal data representation and computational efficiency. The systematic data preparation enhances model performance, generalization, and evaluation accuracy. This dataset establishes a foundation for modeling transition metal sulfide catalytic activity and understanding structure–property relationships.

### 3.2. Fine-Tuning for Performance Enhancement and Model Iterations

The model underwent nine consecutive fine-tuning iterations (1st-FT to 9th-FT) to optimize band gap prediction and predicted stability classification. Unlike traditional models that require strict numerical formatting and explicit feature engineering, the fine-tuned LLM efficiently processes natural language descriptions from diverse sources (research papers, experimental reports, materials databases), automatically extracting relevant features and applying pre-existing domain knowledge to complex material-property relationships.

**Figure 3:**
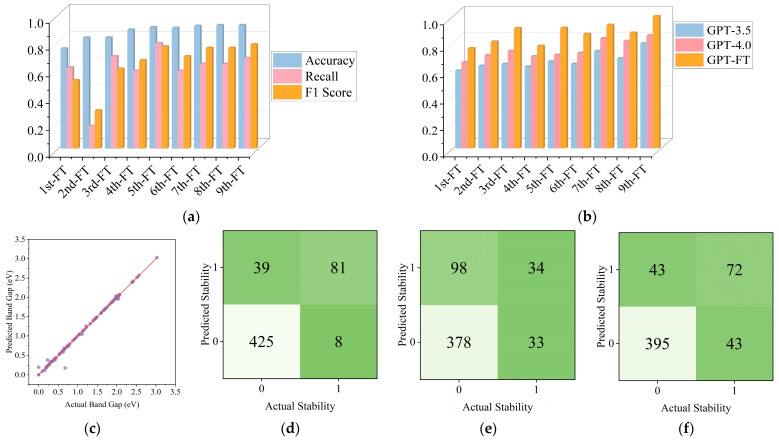
(**a**) Performance metrics for Predicted Stability; (**b**) Comparison of Band Gap prediction accuracy across different models; (**c**) Use a fine-tuned model to predict the prediction accuracy of the Band Gap (purple circles represent individual data points, where each point corresponds to a material with a known actual band gap and the model’s prediction of that band gap, while red line is the ideal prediction line, which represents perfect prediction accuracy); (**d**) Represent the prediction accuracy of Fine-tuned model for Predicted Stability; (**e**) Represent the prediction accuracy of GPT-3.5 for Predicted Stability; (**f**) Represent the prediction accuracy of GPT-4.0 for Predicted Stability.

As shown in Figure 3a, the performance metrics (Accuracy, Recall, F1 Score) gradually improved during the iteration process and reached a peak in the final iterations. Figure 3b compares the performance of GPT-3.5, GPT-4.0, and GPT-FT (fine-tuned) on band gap prediction tasks. Table 1 quantitatively demonstrates this performance difference, with GPT-FT achieving an exceptional R^2^ value of 0.9989, significantly outperforming both GPT-3.5 (R^2^ = 0.7937) and GPT-4.0 (R^2^ = 0.8542). This substantial improvement highlights the effectiveness of our domain-specific fine-tuning approach compared to even the most advanced general-purpose language models. GPT-FT consistently outperforms baseline models, with accuracy exceeding 0.8 across iterations and nearing 1.0 after the ninth fine-tuning. Although GPT-4.0 surpassed GPT-3.5, both lagged significantly behind GPT-FT. All models were improved through gradual fine-tuning iterations, and the performance gap narrowed slightly after the seventh and eighth iterations. Figure 3c confirms strong final results as the data points are almost perfectly spaced from the prediction line. Confusion matrices in Figure 3d–f show notable classification advantages for predicted stability: fine-tuned model F1 > 0.7751, GPT-3.5 F1 > 0.5840, and GPT-4.0 F1 > 0.6261. The LLM’s performance is enhanced due to natural language understanding capabilities, reasoning about incomplete information, and capturing long-range property dependencies, unlike traditional models that are bound by rigid frameworks.

### 3.3. Comparative Analysis of Model Prediction Accuracy

**Figure 4:**
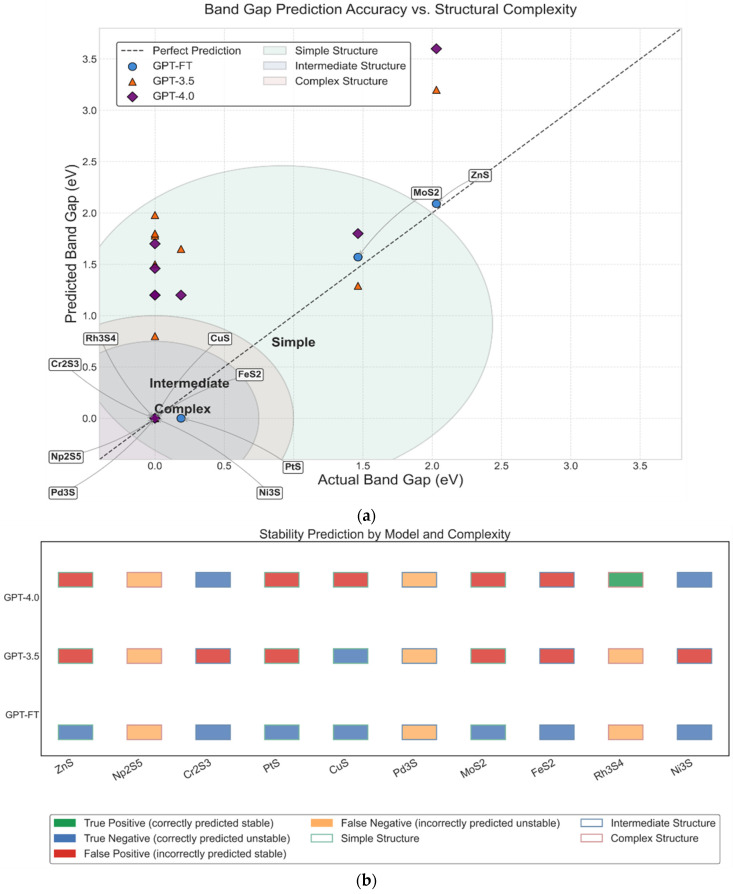
(**a**) Band gap prediction accuracy of GPT-FT, GPT-3.5, and GPT-4.0 across materials of varying structural complexity; (**b**) Predicted stability classification performance of GPT-based models on structurally diverse compounds.

Figure 4 compares the performance of GPT-FT, GPT-3.5, and GPT-4.0 in predicting the band gap and predicted stability of 10 transition metal sulfide compounds with varying structural complexity. In Figure 4a, GPT-FT achieves the best band gap predictions, with the lowest average error (0.035 eV) and accurate identification of all metallic compounds with zero band gap. In contrast, GPT-3.5 and GPT-4.0 exhibit larger errors, frequently misclassifying metallic systems as semiconductors, particularly for compounds with intermediate or complex structures. As shown in Figure 4b, GPT-FT again outperforms the other models in predicting thermodynamic stability, showing minimal errors and strong generalization across structural types. Meanwhile, GPT-3.5 and GPT-4.0 make more frequent misclassifications, especially in complex cases. These consistent performance differences emphasize the advantages of domain-specific fine-tuning in GPT-FT. It can provide a deeper understanding of the physical relationship between structure, electronic properties, and stability, which is lacking in the general model.

Comprehensive performance evaluations were conducted comparing the fine-tuned model against advanced language models and traditional machine learning approaches. The fine-tuned model demonstrated superior data processing flexibility and automated feature extraction capabilities while achieving enhanced performance in band gap prediction and stability classification. This research reveals significant advantages of the fine-tuned model over both advanced language models and traditional machine learning methods. As shown in Figure 3b,c, the optimized GPT-FT achieves band gap prediction R^2^ values approaching 0.9989, RMSE = 0.0252, MSE = 0.0060, through autonomous feature learning and efficient knowledge transfer across material systems, substantially outperforming GPT-3.5 (0.7937) and GPT-4.0 (0.8542).

**Figure 5:**
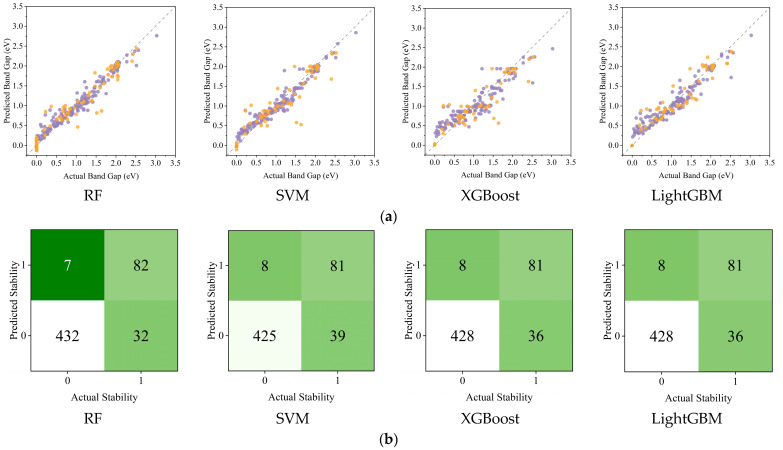
(**a**) Experiment and predict band gap values on training and test sets by four major traditional ML algorithms (the purple points represent the training set data, while the orange points represent the test set data); (**b**) Prediction accuracy of traditional ML algorithms for Predicted Stability.

Traditional machine learning models, despite requiring extensive feature engineering and standardized data preprocessing, achieved varying accuracy in band gap predictions for transition metal sulfides. As shown in Table 2, all four traditional algorithms demonstrated strong performance on both training and testing datasets. RF achieved the highest testing R^2^ value (0.9655) with relatively low RMSE (0.1491), while maintaining consistent performance between training (R^2^ = 0.9713, RMSE = 0.1077) and testing sets, indicating good generalization. LightGBM showed similar consistency with testing R^2^ = 0.9592 and RMSE = 0.1623. XGBoost exhibited the lowest training RMSE (0.0842) but slightly higher testing error (0.1867), suggesting some overfitting. SVM showed the largest gap between training and testing performance, with the highest testing RMSE (0.1951) among all models. While traditional models in Figure 5a, including RF (R^2^ = 0.9660), LightGBM (R^2^ = 0.9592), XGBoost (R^2^ = 0.9460), and SVM (R^2^ = 0.9410), demonstrate competent performance through manual feature engineering, they lack adaptive knowledge transfer capabilities. Figure 5b demonstrates the fine-tuned model’s advantage in stability prediction, maintaining F1 > 0.7751 despite using a compact training dataset, which is crucial for materials research with limited data availability. This adaptive framework allows continuous model improvement through incremental data integration while preserving high performance metrics, eliminating the need for complete model rebuilding common in traditional approaches.

### 3.4. Analysis of Prediction Patterns and Model Improvement

Analysis of prediction failures reveals systematic patterns influenced by material characteristics. Through nine iterative fine-tuning iterations, the GPT-FT model showed remarkable improvement, with band gap prediction R^2^ values increasing from 0.7564 in early iterations to the final 0.9989. This domain-specific optimization proved essential for materials science predictions, conclusively demonstrating that general AI models have limitations in scientific tasks without specialized training. These findings underscore the critical importance of transfer learning for addressing complex materials science challenges, particularly when dealing with novel compound classes constrained by limited experimental data. Through careful data selection and refined techniques for catalytic materials, we have developed an effective approach for materials informatics that efficiently utilizes information from limited but high-quality experimental datasets.

### 3.5. Advantages of Fine-Tuning LLMs for Materials Science Applications

Our experimental results demonstrate clear advantages of fine-tuned language models for transition metal sulfide analysis beyond original performance metrics [40]. The model performs well in problems that are difficult to solve by traditional methods, such as interpreting complex crystallographic information, recognizing relationships between structural features and properties, and transferring knowledge from general materials science to specific transition metal sulfides applications. Unlike traditional models that require complete retraining, our approach enables incremental learning and shows flexibility to data variability commonly found in materials science.

The prediction accuracy is affected by compound structural complexity, and the more complex the system is, the greater the challenge to the prediction accuracy. This model provides better interpretability by identifying the attention mechanism of important structural features, which establishes a foundation for automated materials discovery systems that can accelerate catalyst development through efficient screening [41].

## 4. Conclusions

This research explores LLM fine-tuning for the catalytic activity prediction of transition metal sulfides as an alternative to manual feature prediction and adopts a compact dataset to optimize the fine-tuning process. The fine-tuned GPT-3.5-turbo model achieved R^2^ values of 0.9989 for band gap predictions and an F1 Score > 0.7751 for stability predictions. In performance comparison, the fine-tuned model compares with other machine learning methods: RF (R^2^ = 0.9660, F1 score = 0.8079), SVM (R^2^ = 0.9410, F1 score = 0.7751), XGBoost (R^2^ = 0.9460, F1 score = 0.7864), and LightGBM (R^2^ = 0.9592, F1 score = 0.7864). Although it has demonstrated promising capabilities in automatic feature extraction and transfer learning, the performance of this model mainly depends on the quality and completeness of the training data, especially for complex material systems with limited experimental data. This model currently has the characteristics of direct text data processing, cross-domain knowledge transfer, automatic feature recognition, and stability of material system prediction. However, there are still challenges in effectively integrating multimodal data sources (including experimental images, spectral data, and molecular structures). It is worth noting that although a compact dataset was strategically chosen to optimize fine-tuning efficiency and reduce experimental noise, this approach does not limit the model’s capabilities, as the architecture can easily adapt to dataset expansion and seamlessly integrate new data during subsequent training. Looking forward, the development focus should be concentrated on enhancing multimodal data integration capabilities, developing more effective fine-tuning strategies for different material systems, establishing automated verification protocols, standardizing materials science data formats, and a collaborative platform for continuous model improvement.

## Figures and Tables

**Table 1:** The $R^{2}$ value of GPT-3.5, GPT-4.0, and GPT-FT for the fine-tuned dataset.

	GPT-3.5	GPT-4.0	GPT-FT
R^2^ RMSE	0.7937	0.8542	0.9989
	0.3041	0.2453	0.0252

**Table 2:** The training set results and testing set results of RF, SVM, XGBoost, and LightGBM for the traditional dataset.

	RF	SVM	XGBoost	LightGBM
Train Train R^2^ Train RMSE Test R^2^ Test RMSE	0.9713	0.9533	0.9624	0.9612
	0.1077	0.1874	0.0842	0.1208
	0.9655	0.9410	0.9460	0.9592
	0.1491	0.1951	0.1867	0.1623

## Data Availability

The original contributions presented in this study are included in the article/Supplementary Materials. Further inquiries can be directed to the corresponding author.

## Supplementary Materials

The following supporting information can be downloaded at: https://www.mdpi.com/article/10.3390/ma18163793/s1, Section S1: Model Consistency Improvement Over Training Iterations. Section S2: Code of Models. Section S3: Benchmark results of eight machine-learning algorithms on the transition-metal sulfide dataset. Section S4. (1) Figure S1. The influence of the number of selected features based on Recursive Feature Elimination (RFE) on the cross-validation $R^{2}$ values of four models; (2) Table S1. Cross-validation $R^{2}$ values (average ± standard deviation) of the four models under different feature quantities. Section S5. Impact of Loss-Threshold Settings on Model Performance and Outlier Coverage.
