# Supplementary material for: Fine-Tuned Large Language Models for High-Accuracy Prediction of Band Gap and Stability in Transition Metal Sulfides
Source: Materials (Basel). 2025 Aug 13;18(16):3793. doi: 10.3390/ma18163793 (PMC12387436; doi:10.3390/ma18163793)
Supplement: Supplementary file 1 [file materials-18-03793-s001.zip › materials-3758480-supplementary.pdf]

Supporting information: Fine-Tuned Large Language Models for High-Accuracy Prediction of Band Gap and Stability in Transition Metal Sulfides

Zimo Zhao[1], Lin Hu[2], Honghui Wang[1]

1. Demonstrative Software School, College of Computer Science and Cyber Security, Chengdu University of Technology, Chengdu 610059, China

2. College of Physics and Electronics Engineering, Sichuan Normal University, Chengdu 610101, China

Email: wanghh@cdut.edu.cn

Contents:

Section S1. Model Consistency Improvement Over Training Iterations.

Section S2. Code of Models.

Section S3. Benchmark results of eight machine-learning algorithms on the transition-metal sulfide data set.

Section S4. (1) Figure S4. The influence of the number of selected features based on Recursive Feature Elimination (RFE) on the cross-validation R^2 values of four models; (2) Table S4. Cross-validation R^2 values (average ± standard deviation) of the four models under different feature quantities.

Section S5. Impact of Loss-Threshold Settings on Model Performance and Outlier Coverage.

Section S1. Model Consistency Improvement Over Training Iterations.

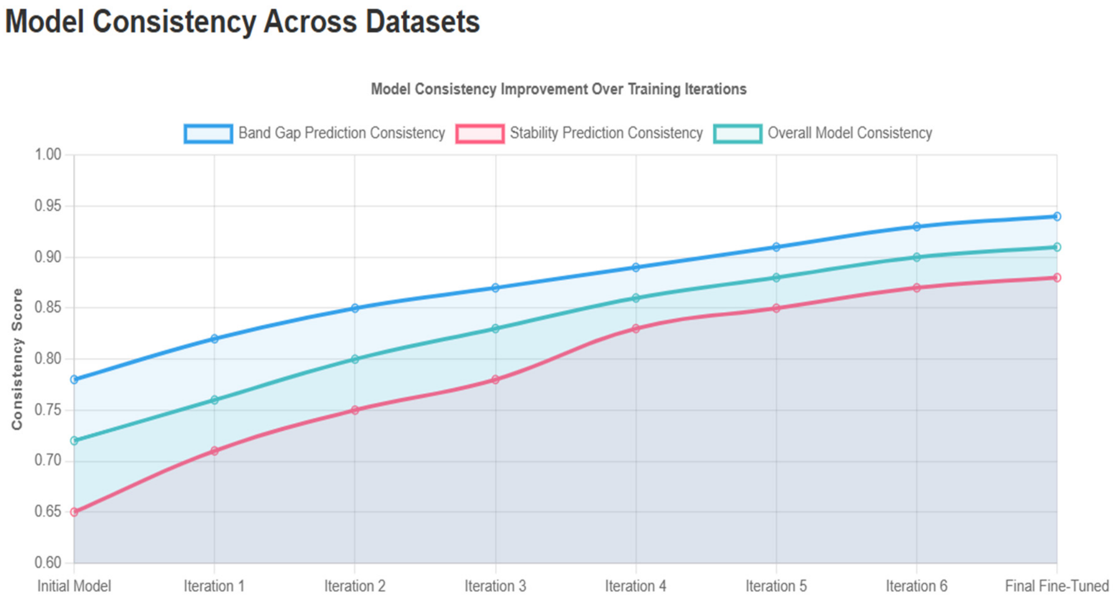

Section S2. Code of Models.

The code and related data sets are in:
https://github.com/Z-Zimo/Supporting---dataset-code/tree/master

1. Fine-tuning model:

Function module	Code snippet
**Training cycles**	n_epochs = 3
**Type of fine-tuning method**	method={<br>"type": "supervised",<br>"supervised": {<br>"hyperparameters": {"n_epochs": n_epochs},<br>},<br>},
**Training model**	model="ft:gpt-3.5-turbo-0125:personal:samantha-test:AtCAfwlb"<br># This is the final fine-tuning model, the initial model is gpt-3.5-turbo
**Early stop standard (maximum waiting time)**	# Maximum waiting time(A parameter in the early shutdown criteria)<br>max_wait_time = 20000<br>start_time = time.time()
**Loss monitoring**	loss_pattern = re.compile(<br>r"Step (\d+)/\d+: training loss=([\d\.]+)"<br>r"(?:, validation loss=([\d\.]+))?"<br>r"(?:, full validation loss=([\d\.]+))?"<br>)<br>steps = []<br>training_losses = []<br>validation_losses = []<br>full_validation_losses = []<br><br>for event in events:<br>message = event.get("message", "")

	match = loss_pattern.search(message)<br>if match:<br>step = int(match.group(1))<br>training_loss = float(match.group(2))<br>validation_loss = float(match.group(3)) if match.group(3) else None<br>full_validation_loss = float(match.group(4)) if match.group(4) else None
**Visualization: Code that draws a loss curve**	if steps and training_losses:<br># Plot a training loss curve<br>plt.figure(figsize=(12, 8))<br>plt.plot(steps, training_losses, marker='o', linestyle='-', color='b', label='Training Loss')<br># Draw a verification loss curve (if any)<br>if any(loss is not None for loss in validation_losses):<br>plt.plot(<br>[s for s, v in zip(steps, validation_losses) if v is not None],<br>[v for v in validation_losses if v is not None],<br>marker='x',<br>linestyle='--',<br>color='r',<br>label='Validation Loss'<br>)<br># Draw a complete verification loss curve (if any)<br>if any(loss is not None for loss in full_validation_losses):<br>plt.plot(<br>[s for s, fv in zip(steps, full_validation_losses) if fv is not None],<br>[fv for fv in full_validation_losses if fv is not None],<br>marker='s',<br>linestyle=':',<br>color='g',<br>label='Full Validation Loss'<br>)<br><br>plt.title('Loss Over Steps')<br>plt.xlabel('Step')<br>plt.ylabel('Loss')

	plt.legend()<br>plt.grid(True)<br>plt.tight_layout()<br>plt.savefig('training_loss_validation.png')<br>plt.show()
**Set a high loss threshold**	training_high_loss_threshold = 0.5<br>high_loss_steps = [step for step, loss in zip(steps, training_losses) if loss > training_high_loss_threshold]<br>print(f"\nHigher than {training_high_loss_threshold} The number of training lost steps: {len(high_loss_steps)}")
	# Extract the data corresponding to the high loss validation step (based on validation loss and full validation loss)<br>validation_high_loss_threshold = 1.0 # Set the verification loss threshold, which can be adjusted as needed<br>high_loss_validation_steps = []<br><br># Collect validation loss > threshold<br>for v_loss in validation_losses:<br>if v_loss is not None and v_loss > validation_high_loss_threshold:<br>high_loss_validation_steps.append(1) # Record a high loss event<br><br># Collect full validation loss > threshold<br>for fv_loss in full_validation_losses:<br>if fv_loss is not None and fv_loss > validation_high_loss_threshold:<br>high_loss_validation_steps.append(1) # Record a high loss event

2. Traditional Models:

(1) RF:

Function module	Code snippet
**Cross validation strategy: 3-fold cross validation**	grid_search = GridSearchCV(multi_output_rf,param_grid,<br>scoring='r2', cv=3, n_jobs=-1, verbose=2,<br>error_score='raise')<br>grid_search.fit(X_train, y_train)
**Random seed: random_state=42**	X_train, X_test, y_train, y_test = train_test_split(X, y, test_size=0.2, random_state=42)
	multi_output_rf =<br>MultiOutputRegressor(RandomForestRegressor(random_state=42))
**Feature selection and dimensionality reduction methods: PCA and RFE**	# Dimensionality reduction using PCA<br>pca = PCA(n_components=0.95)<br>X_reduced = pca.fit_transform(X_scaled)<br># Feature selection using RFE<br>rf = RandomForestRegressor(n_estimators=100, random_state=42)<br>selector = RFE(rf, n_features_to_select=10, step=5)<br>X_selected = selector.fit_transform(X_scaled, y)<br># Feature importance selection<br>rf.fit(X_scaled, y)<br>importances = rf.feature_importances_<br>indices = np.argsort(importances)[-10:]<br>X_selected = X_scaled[:, indices]
**Data preprocessing strategy: standardization, missing value processing**	# Feature scaling<br>scaler = StandardScaler()<br>X_scaled = scaler.fit_transform(X)<br># Impute missing values<br>imputer = SimpleImputer(strategy="constant", fill_value=0)<br>X_scaled = imputer.fit_transform(X_scaled)<br># Replace inf and -inf with NaN in original X (for safety)<br>X.replace([np. inf, -np. inf], np. nan, inplace=True)<br>X.fillna(0, inplace=True)
	# Parameter grid<br>param_grid = {<br>'estimator__n_estimators': [100, 200, 300],<br>'estimator__max_depth': [10, 20, 30],

	'estimator__min_samples_split': [2, 5, 10],<br>'estimator__min_samples_leaf': [1, 2, 4],<br>'estimator__max_features': ['sqrt', 'log2', None]<br>}

(2) SVM:

**Function module**	**Code snippet**
**kernel function: kernel='rbf'**	svr = SVR(kernel='rbf')
**Cross validation strategy: 50% cross validation**	grid_search = GridSearchCV(xgboost_model, param_grid, scoring='r2', cv=5, verbose=2, n_jobs=-1)
**Feature selection: RFE, n_features_to_select=20**	selector_band_gap = RFE(svr, n_features_to_select=20, step=1)<br>X_selected_band_gap = selector_band_gap.fit_transform(X_scaled, y['Band Gap'])<br><br>selector_pred_Stability = RFE(svr, n_features_to_select=20, step=1)<br>X_selected_pred_Stability = selector_pred_Stability.fit_transform(X_scaled, y['Predicted Stability'])
**Data preprocessing: standardized, missing value processing**	X.replace([np.inf, -np.inf], np.nan, inplace=True)<br>X.fillna(0, inplace=True)<br><br>scaler = StandardScaler()<br>X_scaled = scaler.fit_transform(X)
	if y.isnull().values.any():<br>y = y.fillna(0)

(3) XGBoost:

Function module	Code snippet
**Basic parameter**	model = MultiOutputRegressor(XGBRegressor(n_estimators=100, learning_rate=0.1, max_depth=5, random_state=42))
**Training/test set split (80%/20%)**	# Split into training and test sets<br>X_train, X_test, y_train, y_test = train_test_split(X, y, test_size=0.2, random_state=42)
**Evaluation index: R^2, RMSE, MSE**	# Evaluation metrics for training set (Band Gap)<br>r2_band_gap_train = r2_score(y_train['Band Gap'], y_train_pred[:, 0])<br>mse_band_gap_train = mean_squared_error(y_train['Band Gap'], y_train_pred[:, 0])<br>rmse_band_gap_train = np.sqrt(mse_band_gap_train)<br><br># Evaluation metrics for test set (Band Gap)<br>r2_band_gap_test = r2_score(y_test['Band Gap'], y_test_pred[:, 0])<br>mse_band_gap_test = mean_squared_error(y_test['Band Gap'], y_test_pred[:, 0])<br>rmse_band_gap_test = np.sqrt(mse_band_gap_test)

(4) LightGBM:

Function module	Code snippet
**Training cycle 1000, early stop set 100 rounds**	model = lgb.train(<br>best_params,<br>train_data,<br>num_boost_round=1000,<br>valid_sets=[test_data],<br>early_stopping_rounds=100,<br>verbose_eval=False<br>)

**Training/test set split (80%/20%)**	def train_and_evaluate(target_column):<br>y = df[target_column]<br>X_train, X_test, y_train, y_test = train_test_split(X, y, test_size=0.2, random_state=42)
**Bayesian optimization hyperparameters**	bounds = {<br>'num_leaves': (20, 100),<br>'learning_rate': (0.001, 0.1),<br>'feature_fraction': (0.5, 1.0),<br>'bagging_fraction': (0.5, 1.0),<br>'max_depth': (5, 20),<br>'min_data_in_leaf': (10, 100),<br>'lambda_l1': (0, 10),<br>'lambda_l2': (0, 10),<br>}<br>optimizer = BayesianOptimization(f=lgb_cv, pbounds=bounds, random_state=42)<br>optimizer.maximize(init_points=10, n_iter=50)

Section S3. Benchmark results of eight machine-learning algorithms on the transition-metal sulfide data set.

**Algorithm models**	$\mathbf{R^2}$	**RMSE(eV)**
RF	0.9655	0.1491
SVM	0.9410	0.1951
XGBoost	0.9460	0.1867
LightGBM	0.9592	0.1623
MLP-Regressor	0.9382	0.2017
GradientBoosting	0.9291	0.2137
k-NN	0.9123	0.2315
Gaussian Process	0.9246	0.2194

Section S4.

(1) **Figure S1. The influence of the number of selected features based on Recursive Feature Elimination (RFE) on the cross-validation R^2 values of four models**

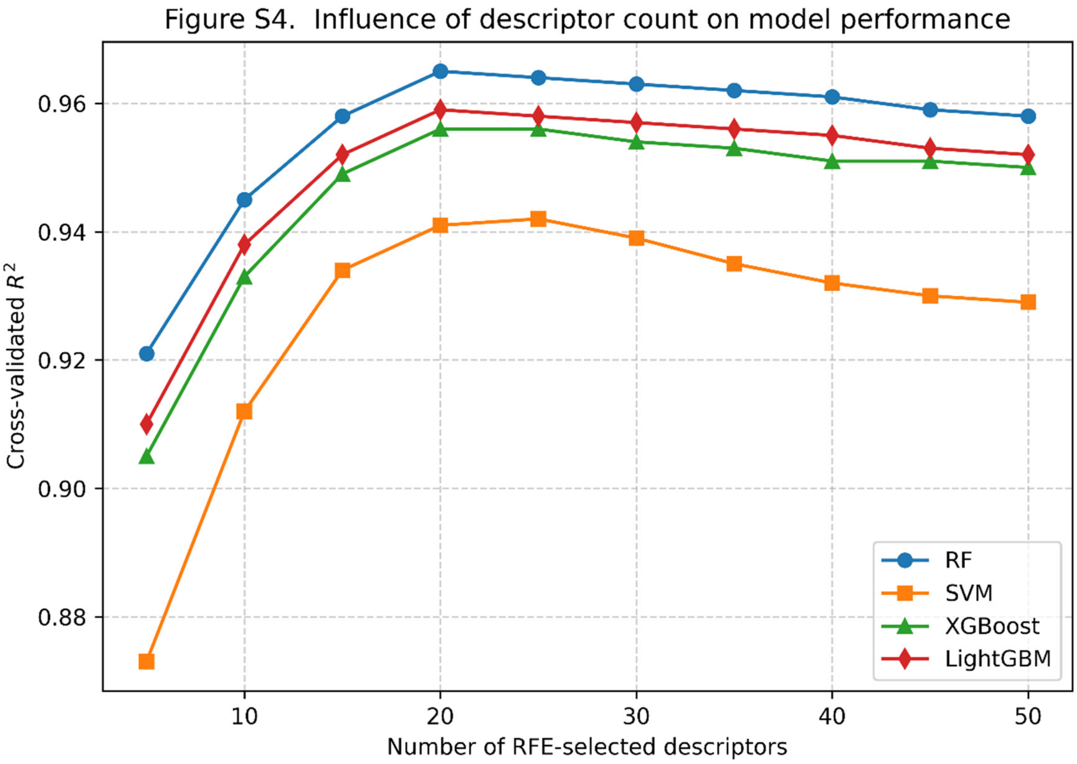

(2) Table S1. Cross-validation R^2 values (average ± standard deviation) of the four models under different feature quantities

Descriptors	RF	SVM	XGBoost	LightGBM
5	0.921±0.015	0.873 ± 0.020	0.905 ± 0.018	0.910 ± 0.017
10	0.945 ± 0.012	0.912 ± 0.018	0.933 ± 0.015	0.938 ± 0.014
15	0.958 ± 0.010	0.934 ± 0.014	0.949 ± 0.013	0.952 ± 0.011
**20**	**0.965 ± 0.009**	**0.941 ± 0.012**	**0.956 ± 0.011**	**0.959 ± 0.010**
25	0.964 ± 0.011	0.942 ± 0.014	0.956 ± 0.012	0.958 ± 0.011
30	0.963 ± 0.013	0.939 ± 0.017	0.954 ± 0.013	0.957 ± 0.012
35	0.962 ± 0.013	0.935 ± 0.019	0.953 ± 0.013	0.956 ± 0.013
40	0.961 ± 0.014	0.932 ± 0.020	0.951 ± 0.014	0.955 ± 0.013
45	0.959 ± 0.015	0.930 ± 0.021	0.951 ± 0.015	0.953 ± 0.014
50	0.958 ± 0.016	0.929 ± 0.022	0.950 ± 0.016	0.952 ± 0.015

Section S5. Impact of Loss-Threshold Settings on Model Performance and Outlier Coverage.

Threshold setting (train / val)	Training loss coverage	Verified loss coverage rate	9th-FT R^2	9th-FT F1
0.4/0.8	97.1%	97.4%	0.9988	0.7750
**0.5/1.0**	**95.0%**	**95.1%**	**0.9989**	**0.7751**
0.6 / 1.2	92.4 %	92.7 %	0.9988	0.7748
